# Chemical and Functional Aspects of Posttranslational Modification of Proteins

**Published:** 2009-10

**Authors:** D.G. Knorre, N.V. Kudryashova, T.S. Godovikova

**Affiliations:** 1 Institute of Chemical Biology and Fundamental Medicine, Siberian Branch, Russian Academy of Sciences

## Abstract

This paper reviews the chemical and functional aspects of the posttranslational modifications of proteins, which are achieved by the addition of various groups to the side chain of the amino acid residue backbone of proteins. It describes the main prosthetic groups and the interaction of these groups and the apoenzyme in the process of catalysis, using pyridoxal catalysis as an example. Much attention is paid to the role of posttranslational modification of proteins in the regulation of biochemical processes in live organisms, and especially to the role of protein kinases and their respective phosphotases. Methylation and acetylation reactions and their role in the "histone code", which regulates genome expression on the transcription level, are also reviewed. This paper also describes the modification of proteins by large hydrophobic residues and their role in the function of membrane-associated proteins. Much attention is paid to the glycosylation of proteins, which leads to the formation of glycoproteins. We also describe the main non-enzymatic protein modifications such as glycation, homocysteination, and desamida-tion of amide residues in dibasic acids.

## INTRODUCTION

Template biosynthesis of polypeptide chains on ribosomes most often does not immediately produce a fully functional protein. The newly formed polypeptide chain must undergo certain chemical modifications outside the ribosome. These modifications are most often driven by enzymes and take place after all the information supplied by the template RNA (mRNA) has been read, that is after mRNA translation: thus, these additional processes are called posttranslational modifications.

Posttranslational protein modification processes can be divided into two main groups. The first group unites proteolytic processes, which are mainly cleavages of certain peptide bonds, resulting in the removal of some of the formed polypeptide fragments. The second group consists of the processes that modify the side chains of the amino acid residues and usually do not interfere with the polypeptide backbone. The chemical nature and function of these modifications is diverse. Moreover, each type of modification is characteristic of certain groups of amino acid residues. The result of these processes is that the proteome of the cell or organism consists of several orders more components than there are genes encoding these components of the proteome. This paper is a review of the second group of posttranslational protein modifications.

There are four main groups of protein functions that require posttranslational modification of amino acid residue side chains. The functional activity of a wide number of proteins requires the presence of certain prosthetic groups covalently bound to the polypeptide chain. These are most often complex organic molecules which take a direct part in the protein's activity. The transformation of inactive apoproteins into enzymes is one of these modifications. Another important group of posttranslational modifications regulates biochemical processes by varying (sometimes switching on and off) enzymatic activity. Another large group of modifications are protein tags, which provide intracellular localization of proteins, including marking the proteins for transport to the proteasome, where they will be hydrolysed and proteolysed. And finally, some posttranslational modifications directly or indirectly influence the spatial structure of newly synthesized proteins.

## MODIFICATION OF PROTEINS BY ADDITION OF PROSTHETIC GROUPS

In some cases, the last step in the biosynthesis of a functional protein is the covalent binding of a prosthetic group, which forms part of the active site [1, 2]. Table 1 shows the structural formulas of side chain modification products after the covalent binding of certain cofactors to proteins, as well as the types of reactions in which the corresponding prosthetic groups take part.

**Table 1:** The main prosthetic groups involved in biocatalytic reactions

Coenzyme name	Structure of prosthetic group derivative	Classes of enzymes. Type of reaction, which involves the prosthetic group
Biotin	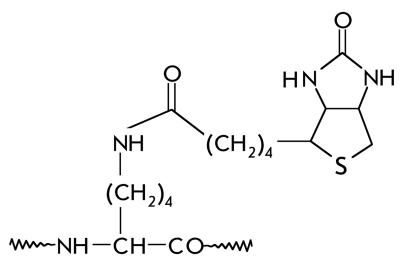	Carboxylases. E.C. 6.4.1.2; 6.4.1.3. Carboxylation. Transfer of a single carbon fragment (CO_2_) onto acetyl- CoA, propionyl-CoA, and other organic molecules
Lipoate	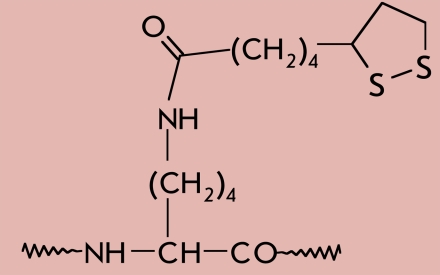	Acyltransferases. E.C. 2.3.1.12. Reduction-oxidation. Transfer of carbon fragments onto CoA via reductive acylation of lipoamide during oxidative decarboxylation of α-ketoacids.
Panthotenate	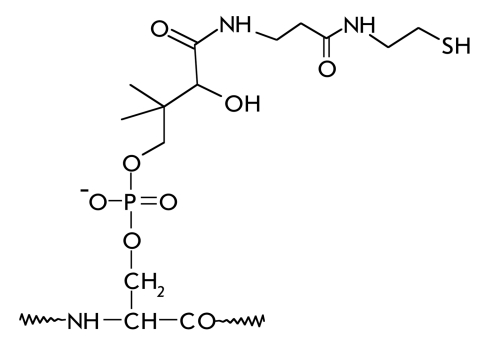	Acyltransferases. E.C. 2.3.1.85. Transacylation. Transfer of an acyl fragment from one enzyme of a multi-enzyme complex to another.
Pyridoxal phospate	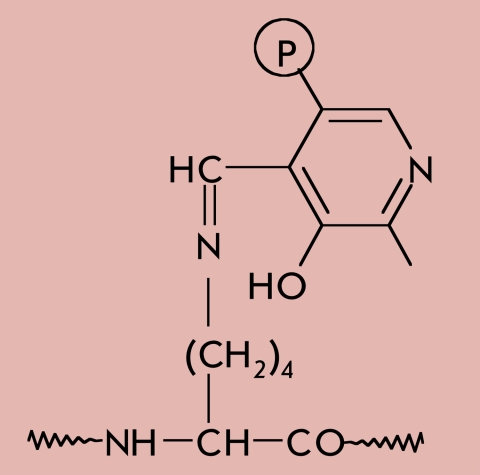	Aminotransferases. E.C.2.6.1. Transamination of amino acids.
Heme	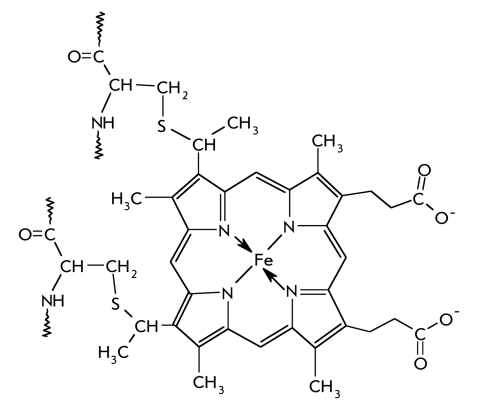	cytochrome c oxidase. E.C. 1.9.3.1. Reduction-oxidation. Transfer of electrons on the mitochondrial membrane during oxidative phosphorylation.
FAD	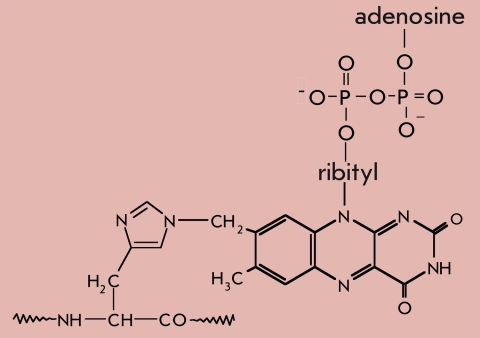	Oxidoreductases. E.C. 1.3.99.1. Reduction-oxidation. Oxidation of the -CH_2_-CH_2_- group down to trans-CH=CH-

Most of the listed prosthetic groups remain covalently bound to the apoenzyme through the whole catalytic process. The only exceptions are pyridoxal enzymes, which experience a demodification of the protein during catalysis; namely, a conversion of the bond between pyridoxal phosphate and the lysine amino group of the apoenzyme into the bond between the coenzyme and the substrate amino acid. A dynamic model of the reaction processes, catalyzed by transaminases, was suggested by M.Y. Karpeisky and V.I. Ivanov in 1969 [3]. Later [4], the authors suggested that the phosphate and methyl groups of the coenzyme act as a sort of axis around which pyridoxal can rotate, thus forming either enzyme-imine or substrate-imine covalent compounds. X-ray analysis data confirmed and detailed the conclusion about pyridoxal phosphate multi-point binding.

**Fig. 1.**
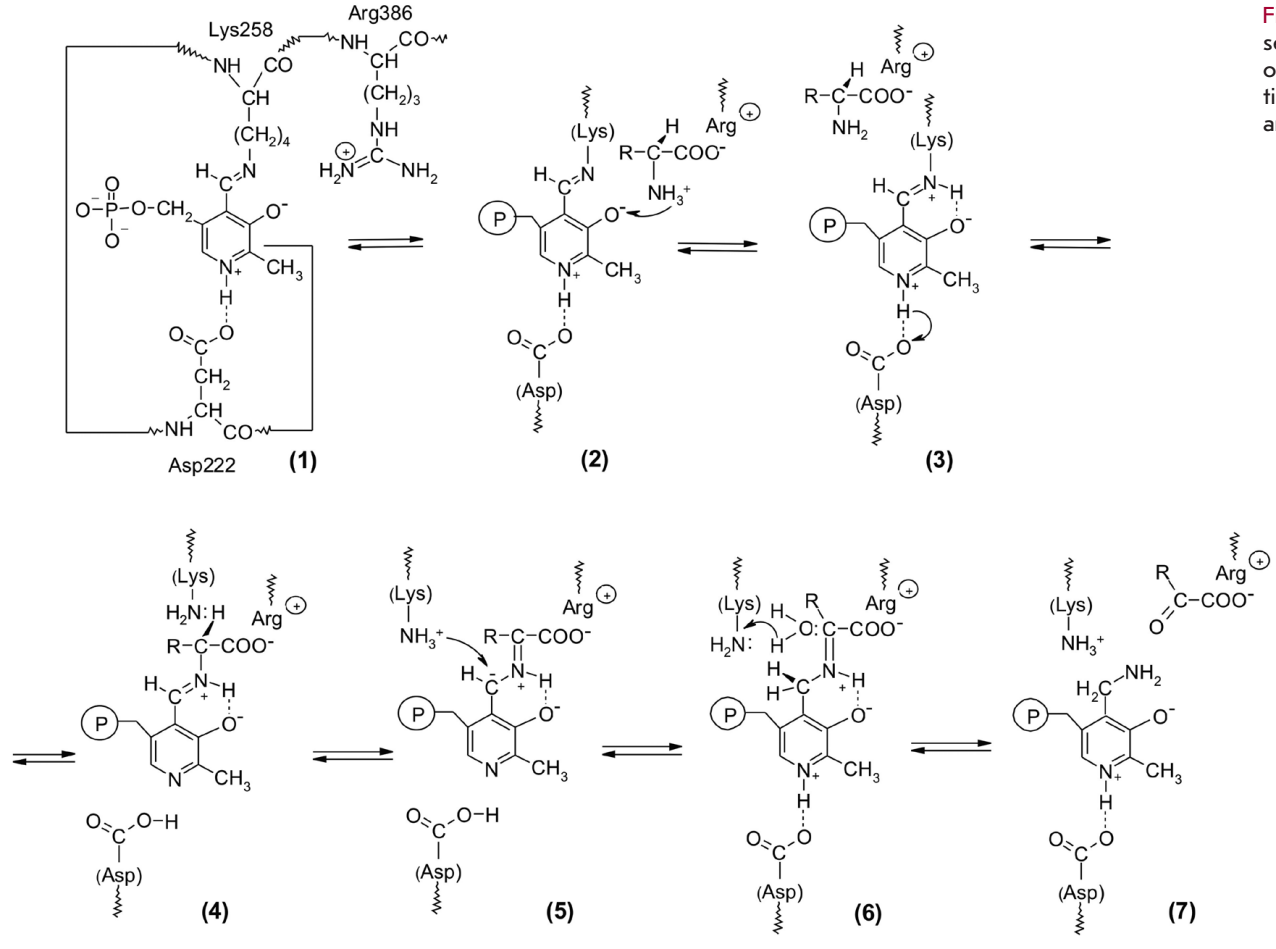
A schematic representation of the first stage of the transamination reaction catalyzed by aspartate aminotransferase

Aspartate aminotransferase (C.E. 2.6.1.1), which catalyses the transamination of oxalacetate and glutamate, can be used to illustrate the mechanism of action of pyridoxal enzymes (Fig. 1).

The coenzyme of the transaminase is not present as a free aldehyde, rather it is an intramolecular aldimine with the lysine side chain amino-group (Lys-258). The enzyme-bound imine assures the high rate of the reaction, as compared to the free pyridoxal phosphate [2-4]. It is this structure that causes the higher activity of imines as compared to aldehydes. The more basic nitrogen of imines is protonated much more efficiently than the carbonyl group oxygen atom (Fig. 1, (3)). The resulting transfer of the proton from the Alpha;-NH_3_^+^-group of the substrate to the atom of N-aldimine pyridoxalphosphate creates the required cationic form of the coenzyme and, simultaneously, a deprotonated amino acid (3). Moreover, the imine carbon is more electrophilic than the carbonyl one, which means that it is more easily attacked by the deprotonated amino group of the Alpha;-amino acid (Fig. 1, (4)). An increase of the electrophilicity of this site is also achieved by the interaction of heterocycle nitrogen with an aspartate residue of the enzyme (hydrogen bond with Asp-222). Thus, the transitional imine-enzyme promotes the rapid formation of a transient bond between the substrate and the coenzyme.

The described example of pyridoxal catalysis illustrates the fact that the apoenzyme plays as important a role in catalysis as the prosthetic group; that is, the former cannot simply be called a carrier of the catalytic group. This is also the case for other prosthetic groups.

## REGULATION OF ENZYME ACTIVITY BY PHOSPHORYLATION

**Fig. 2.**
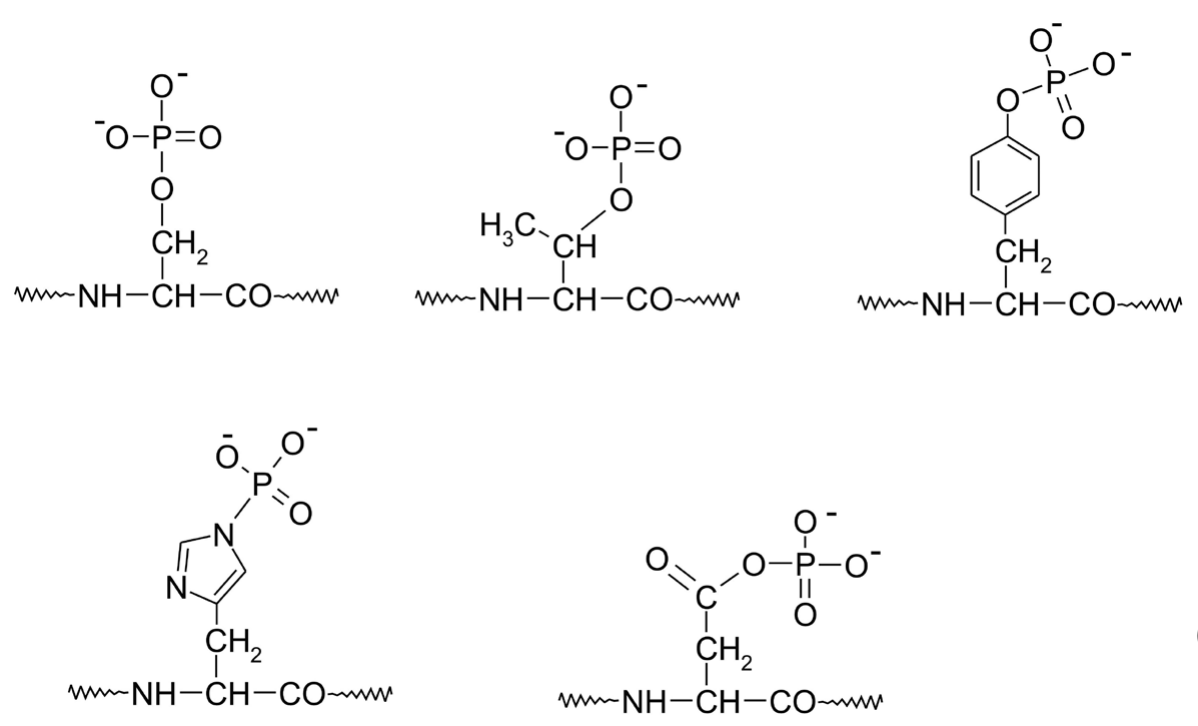
The structure of phosphorylated amino acid fragments

The central role in reactions responsible for rearrangement of all intracellular processes eventually signaling either cell division or cell death is played by a large group of enzymes called protein kinases (phosphotransferases, EC 2.7.). These enzymes can add phosphate groups to the side chains of amino acids in various proteins [5-12]. γ-phosphate ATP is the donor of a phosphate group in all such reactions. Kinases are grouped according to the amino acid to which they add the phosphate into tyrosine kinases (E.C. 2.7.10.2) and serine/threonine kinases (E.C. 2.7.11.1) [5]. Also, histidine kinases are often found in bacteria, plants, and fungi. The latter enzymes function in a two-step signal transduction system [13]. The inorganic phosphate residue, which is attached to a histidine in the enzyme itself, is then transferred onto an aspartate residue in the target protein. Phosphorylation of the aspartate results in further signal transduction [13]. Figure 2 shows the structures of amino acid phosphorylation products in proteins [1].

**Fig. 3.**
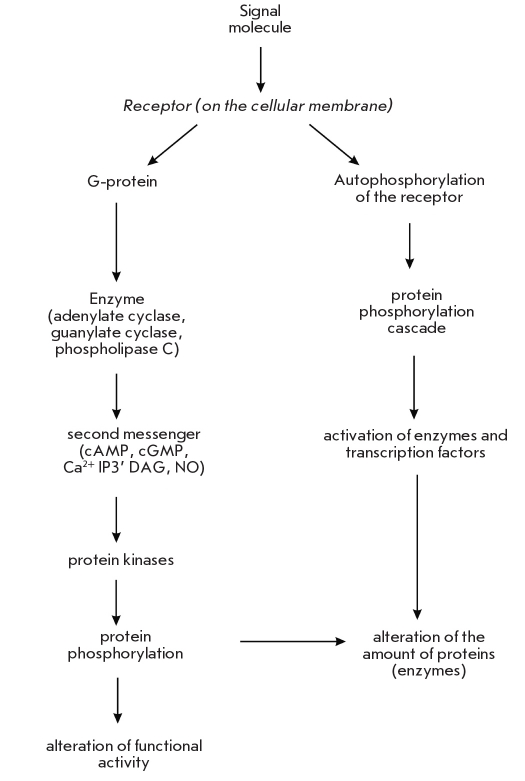
The basic stages of signal transduction via protein phosphorylation. IF - inositoltriphosphate, DAG - diacylglycerine

Concerted regulation of interactions in a multicellular organism is achieved by the release of specialized molecules (hormones, cytokines, etc.) which activate a signaling cascade in target cells. In cases where the signal causes alterations in the expression level of certain genes, the final links in the signaling chains are transcription factors [14-18]. Target cells can identify the signaling molecule amongst a multitude of others with the help of a receptor protein present on the target cell. This protein receptor has a specific binding site for the appropriate signaling molecule. Some receptors are localized on the surface of the cellular membrane, while others are intracellular receptors and are localized in the cytoplasm or inside the nucleus. A schematic representation of the main stages of, for example, hormone signal transduction via membrane receptors is presented in Fig. 3. At some of these stages, the activity of enzymes is regulated by phosphorylation.

Membrane receptors can be divided into three functionally distinct structural regions. The first domain (the recognition domain) is situated in the N-terminal region of the polypeptide chain and is located on the outside of the cellular membrane. This region carries glycosylated sites and recognizes and binds the signaling molecule. The second domain is the transmembrane domain. In some receptors, which are coupled to G-proteins, this domain consists of 7 tightly packed Alpha;-helix polypeptide regions. Another type of receptor has a transmembrane domain that consists of a single Alpha;-helix region. The third (cytoplasmic) domain creates a chemical signal inside the cell, which couples the binding of a signal molecule (a ligand) to a specific intracellular signal.

The cytoplasmic regions of a number of receptors which face onto the inner side of the membrane exhibit tyrosine kinase activity. For instance, the binding of the insulin hormone to its membrane receptor, which is a tyrosine kinase and has a phosphorylation site, causes autophosphorylation and leads to phosphorylation of the receptor's substrates and also of other proteins [10]. The epidermal growth factor receptor (EGFR) belongs to a family of growth factor receptors which bind protein ligands and also exhibit tyrosine kinase activity [14]. After binding the appropriate ligand, the receptor forms a dimer, five tyrosine residues are autophosphorylated on the C-terminus of the receptor, and the protein acquires intracellular tyrosine kinase activity. Further EGFR activity is involved in the initiation of the signal transduction cascade, which includes the activation of mitogen-activated protein kinases, protein kinase B, JNK (Jun N-terminal kinase), or Stress Activated Protein Kinase (SAPK) - the so-called MAP-kinase family. This promotes DNA synthesis and proliferation [11, 12, 18-20].

Cytoplasmic domains of other receptors (somatotropin, prolactin, cytokines, etc.) do not exhibit tyrosine kinase activity themselves but are instead associated with other cytoplasmic protein kinases (the so-called «Janus kinases» or JAK family kinases), which phosphorylate the receptors and thus activate them [11, 18]. The defining feature of Janus kinases among all the other mammalian tyrosine kinases is their tandem kinase (JH1) and pseudokinase (JH2) domains. The latter is the cause for the name "Janus kinase," since they are the only mammalian tyrosine kinases with a pseudokinase domain; thus, they have two "faces" just as the two-faced god Janus. The pseudokinase domain, though it is very similar to kinase domains, does not possess any of the residues responsible for phosphotransferase activity. Apparently, the function of this domain is the regulation of catalytic activity.

**Fig. 4.**
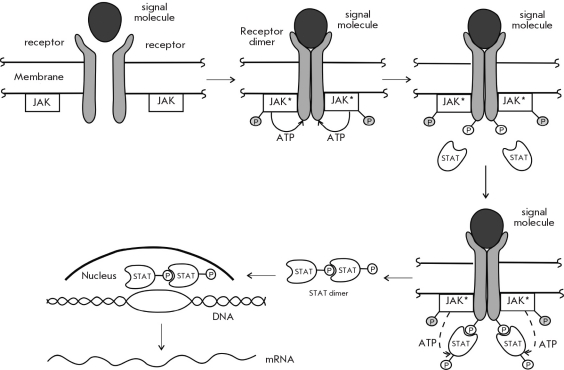
A schematic representation of signal transduction via JAK-associated membrane receptors

Binding of the signaling molecule by a receptor is thought to activate signaling via homo- and heterodimerization of the receptor subunits, which then bind to Janus kinases. This leads to autophosphorylation of the kinases and increases their catalytic activity. The activated Janus kinases phosphorylate tyrosine residues in the subunits of the receptor, which allows the receptor to bind other proteins, for instance the Signal Transducer and Activator of Transcription proteins (STAT). These STAT proteins are then phosphorylated by the Janus kinases, form dimers, and are transported into the nucleus, where they bind specific DNA motifs, thus regulating transcription (Fig. 4).

Mitogen-activated kinases (MAPK, E.C. 2.7.11.24) respond to extracellular stimuli (mitogens) and regulate a range of cellular processes (gene expression, cell division, differentiation, and apoptosis) [11, 17-20]. This MAP signal cascade is conservative in eukaryotes, from yeast to mammals.

The activity of serine/threonine protein kinases is influenced by a number of factors, for instance damage to DNA, and also a range of chemical signals, including cAMP, cGMP, diacylglycerol, and Ca^2+^ calmodulin [5, 8, 21-24]. This type of protein kinases phosphorylates serine or threonine residues in consensus sequences, which form a phosphoacceptor site. This amino acid sequence in the substrate molecule allows contact between the catalytic groove of the protein kinase with the phosphoacceptor site, which creates kinase specificity not towards a certain substrate but towards a certain family of proteins sharing the same consensus sequence. While the catalytic domains of the protein kinases are highly conservative, the recognition sites vary, which allows the recognition of various substrates. Protein kinases A, B, C, G, calmodulin-dependent protein kinases, etc. are all regulated by hormone signal second messengers.

The phosphorylation reaction can take place not only at a single site in the protein molecule, but also at multiple sites, which causes the phosphorylation of the functional groups of various amino acid residues [25-28]. Multiple phosphorylation is characteristic of several enzymes; for instance eukaryotic RNA polymerase II (E.C. 2.7.7.6) [28]. The C-terminus of this enzyme's major subunit carries a large number (52 for mammals, 26-27 for yeast) of repeated heptapeptide consensus sequences (Tyr-Ser-Pro-Thr-Ser-Pro-Ser). Multiple phosphorylation of these repeats at the serine and threonine residues enhances the binding of a large number of transcription elongation factors and their associated proteins. This is a vital step in conversion of the enzymatic transcription preinitiation complex into a stable elongation complex [29], which allows the RNA polymerase to move along the chromatin DNA.

## PROTEIN ACETYLATION

One of the widely spread types of posttranslational modification that plays an important role in living organisms is acetylation [30-38]. The reaction takes place at the ε-aminogroups of lysine residues, and acetyl coenzyme A acts as a donor of acetyl groups. The positive charge of the amino group disappears after this reaction, causing a redistribution of charge in the whole protein molecule, and also increasing the hydrophobicity and size of the modified amino acid's side chain. Among other things, histones use this as a binding signal for transcription factors and associated proteins, i.e. transcription initiation. A very important feature of the proteins that can be acetylated is a so-called bromodomain, a conservative 110 amino acid module [30, 31].

The acetylation process has been well studied on histone proteins [32-38]. Selective acetylation of several lysine residues creates specific chromatin affinity towards certain transcription factors, which predetermines which genes will be expressed. This is why the distribution of acetylation sites between histones and among their amino acid residues is an important factor in the regulation of chromatin expression and is usually considered as one of the elements of the "histone code," which governs the above-mentioned process. In general, the "histone code" includes the whole range of amino acid modifications in the N- and C-terminal sequences of histones (phosphorylation, acetylation, methylation, and ADP-ribosylation), which determines the functional status of the gene with respect to replication and transcription [33-38].

Various forms of the histone acetyltransferase (E.C. 2.3.1.48) catalyze the acetylation of lysine residues located at specific positions in the protein molecule. For instance, the octamer core of a nucleosome, which consists of two copies of H2A, H2B, H3, and H4 histones, contains 30 conservative lysine residues available for acetylation in the N-terminal domains of the proteins (residues in positions 5 and 9 in H2A; residues 5, 12, 15 and 20 in H2B; residues 9, 14, 18, 23 and 27 in H3; and residues 5, 8, 12 and 16 in H4) [39]. Since the number of modified amino acid residues and their size can vary, this creates a multitude of combinations for acetylated residue distribution, which plays an important role in chromatin function. For instance, acetylation of Lys-18 in Saccharomyces cerevisiae yeast histone H3 is the main indicator of active chromatin transcription. This modified residue binds the largest number of transcription factors. Activation of β-interferon genes in humans requires acetylation of Lys-8 in the H4 histone and Lys-14 in the H3 histone [39].

It was discovered that acetylation of lysine residues in the C-terminal domains of proteins protects the protein from modification by ubiquitin, thus increasing the lifespan and active functioning time of this protein. [40].

## ACYLATION OF PROTEINS BY HIGHER FATTY ACID RESIDUES

The most widespread modifications by addition of fatty acid residues are myristoylation, which is the addition of a CH_3_-(CH_2_)_12_-CO- residue to the amino group of an N-terminal glycine [1, 41, 42], and palmitoylation, which is the addition of a CH_3_-(CH_2_)_14_-CO- residue at the SH-group of a cysteine residue [1, 43, 44]. In both cases, the acylation is accomplished by the appropriate acyl coenzyme A, which is produced during oxidative decay of longer fatty acids.

An N-terminal glycine residue [42, 45] appears in proteins after the N-terminal methionine residue, used to signal the start of translation, is cleaved away. Addition of the myristil group is catalyzed by the myristoyl CoA: protein N-myristoyltransferase (E.C. 2.3.1. 97) [46, 47]. The formation of an amide bond between glycine and myristate is an irreversible process. Introduction of the myristoyl residue alters the lypophilic qualities of the protein molecule and promotes weak and reversible interactions of the protein with the phospholipid membranes or hydrophobic domains of other proteins. Such an interaction is vital for cell signaling, apoptosis, and extracellular protein transport activities. Protein kinase A and GAG, one of the main structural proteins of HIV, are examples of myristoylated proteins [45, 48]. Usually, modification by myristic acid acts in conjunction with other protein regulatory mechanisms.

Often, myristoylation of the N-terminal glycine is followed by addition of a palmitic acid residue to a cysteine residue, thus forming a thioester bond [1, 43, 45, 49]. Unlike myristoylation, this modification is reversible: there are several enzymatic mechanisms that catalyze palmitoylation of cysteine residues, as well as their depalmitoylation [50].

Introducing a palmitinic acid residue has the same results as glycine modification by myristate, and the lypophilicity of the protein molecule increases. This enhances the interactions with membranes and promotes transport through them, while the possibility of the reverse depalmitoylation reaction allows the regulation of the protein activity on various stages of the cell cycle and cell signaling. Palmitoylation is usually seen in proteins that participate in signaling: G-proteins (small G-proteins from the Ras-family, Alpha;-subunit of heterotrimeric G-proteins) and non-receptor tyrosine kinases of the Src-family (Fyn, Lck) [43, 45, 47, 51].

## PROTEIN UBIQUITINYLATION

Acylation of proteins by the activated C-terminal carboxyl group of glycine in ubiquitin, an 8kDa peptide consisting of 76 amino acid residues, is of great biological importance [52-59]. The main, although not the only, purpose of this reaction is the marking of proteins for degradation. These include various damaged proteins, as well as ordinary proteins which fulfill their functions in certain phases of the cell cycle and whose activity is unfavorable during other phases.

Conjugation of the target protein and ubiquitin is a three-stage process. The first stage is the activation of the carboxyl group of ubiquitin, performed by the ubiquitin-activating enzyme E1 using ATP, thus forming ubiquitinyl-AMP. The second stage is the transfer of the ubiquitin residue onto the SH-group of the ubiquitin-transporting protein E2. In the third stage, the ubiquitin-protein ligase E3 catalyses the transfer of ubiquitinyl residue onto the protein substrate, forming an amide bond between the C-teminal glycine of ubiquitin (G76) and a lysine residue in the target protein (substrate). A thus-modified protein is a target for proteolysis in proteasomes or lysosomes [57].

Whereas E1 is the single such enzyme in the cell, E2 has 20-40 isoforms, and the E3 enzyme has hundreds of isoenzymes, which differ by the nature of the protein substrate. Preliminary modification of the target protein is often needed in order for the E3 enzyme to recognize its substrate (phosphorylation (Ser/Thr, Tyr), hydroxylation (Pro), glycosylation (Asn), and N-terminal aminoacylation) [54].

**Fig. 5.**
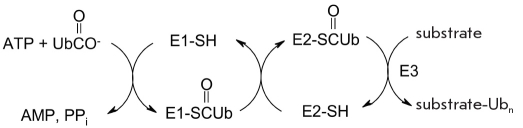
Addition of an ubiquitin residue (residues) onto a substrate protein. E1-SH - ubiquitin-activating enzyme, E2-SH - ubiquitin transport protein, E3 - ubiquitin-protein ligase. Ub - ubiquitin residue

**Fig. 6.**
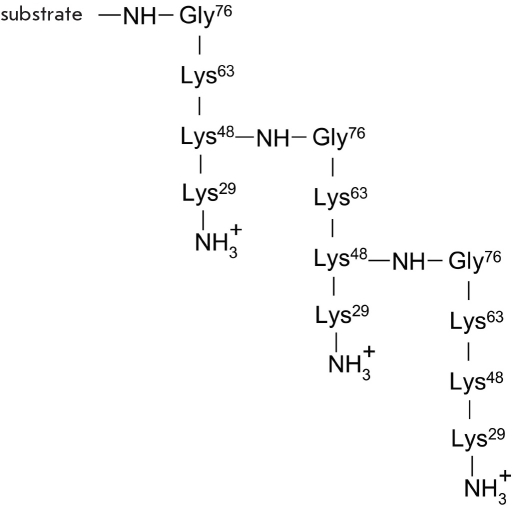
A tandem of several ubiquitin residues bound to the substrate. The numbers refer to the amino acid residues which take part in the modification of the substrate (Gly76) and formation of the tandem (Gly76 and Lys48)

The target protein molecule can be modified by one or several molecules of ubiquitin. The scheme (Fig. 5) denotes such a product as substrate-Ub_n_. Polyubiquitinylation of the substrate involves the acylation of the ubiquitin fragment already bonded to the target protein (Lys-29, Lys-48 or Lys-63) with the C-terminal glycine residue of the other ubiquitin molecule [53, 60-63]. The formation of the ubiquitin-protein covalent adduct does not interfere with the conjugation of the above-named lysine residues with another ubiquitin; thus, this process eventually leads to polyubiquitinylation of the substrate protein. (Fig. 6).

The degree to which the conjugate has been ubiquitylated defines its biological function. Thus, effective proteasome degradation of proteins requires tetraubiquitinylation at Lys29 or Lys48, depending on the target protein. Misfolded proteins and the majority of short-lived proteins form tandem chains of ubiquitin residues connected by bonds at Lys48 [59]. Monoubiquitinylation usually takes place on random multiple lysine residues in the target protein. This happens during the metaphase anaphase transition in mitosis, when metaphase proteins need to be "switched off." Monoubiquitylation of the human H2B is required for the methylation of histone H3, which in turn is very important for chromatin remodeling and for the transcription activation of "silent genes" [35]. Tandems of several ubiquitin residues connected via Lys63 and bonded with PCNA (Proliferating Cell Nuclear Antigen) play in important role in postreplicative DNA reparation [59, 61].

Curently, several ubiquitin-like proteins (ULP) are known, and they are all grouped into the ubiquitin family including ubiquitin itself, Nedd8, Sumo, Fat10, ISG15, Urm1, Hub1, etc. [53, 56-59, 62, 64]. These proteins are variously homologous to ubiquitin in their amino acid sequence and share a similar spatial structure. A large number of ULP in cells indicates their involvement in a wide range of different cellular processes. Thus, Sumo is involved in nuclear transport, transcription regulation and chromosome segregation; ISG15 is part of the immune response cascade; Nedd8 is involved in the meiosis-mitosis switch; and Urm1 is implicated in cellular growth at elevated temperatures [59].

Chaperones interact with newly synthesized, misfolded polypeptides and act as cofactors for ubiquitinylation enzymes, since they possess an ubiquitin-recognition domain. After the target protein has been tagged by ubiquitin, the chaperones escort the ubiquitinylated protein into the proteasome, where they dissociate from the protein complex. The ubiquitin chains are unbound, and the target protein is denaturated via an ATP-dependent process and then broken down into short peptides by proteases.

## PROTEIN ALKYLATION

**Fig. 7.**
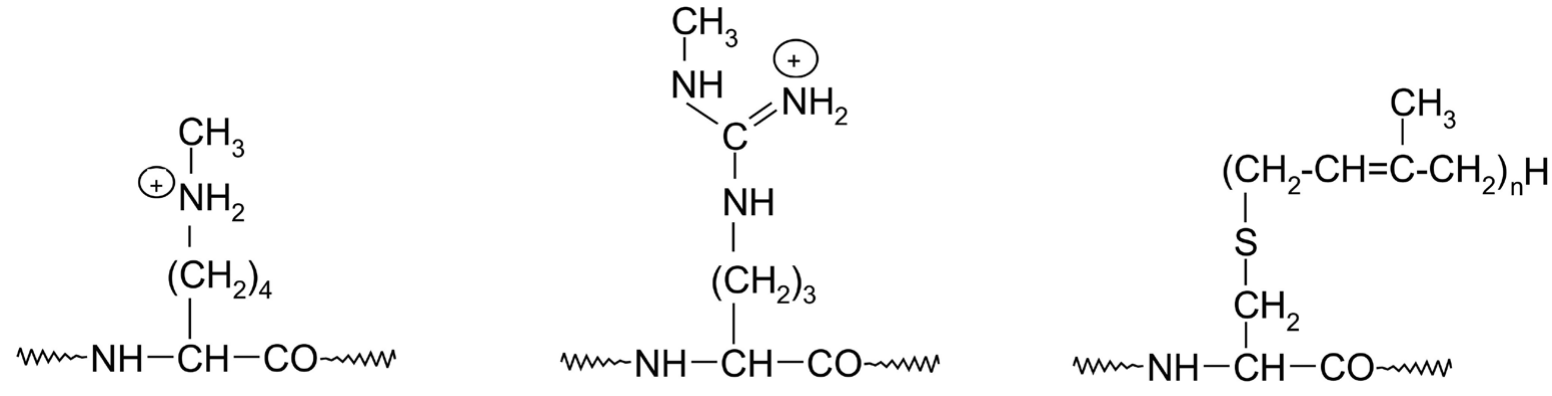
Structures of alkylated amino acid side chains in proteins

Anoter often-seen posttranslational modification is alkylation. This type of modification includes the methylation of lysine and arginine residues [26, 30, 33-38, 39, 65-72] and prenylation (addition of pharnesyl and geranyl-geranyl moieties to cysteine side chains) [47, 73-80] (Fig. 7).

**Fig. 8.**
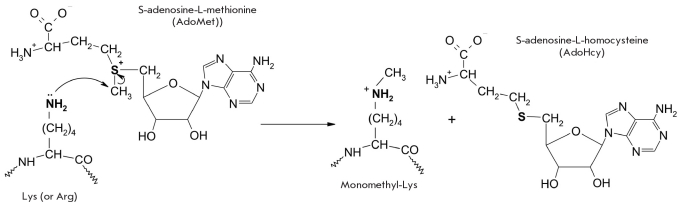
Methylation of lysine residues by methyltransferases

Protein methylation in living organisms is catalyzed by methyltransferases [1, 65, 67] and involves the transfer of a CH_3_-group from S-adenosylmethionine according to the depicted reaction (Fig. 8).

Lysine can form mono-, di- and trimethyllysines in methyltransferase-catalyzed reactions, while arginine can form mono- and dimethylarginines [65]. These compounds differ by size and hydrophobicity from the original residue.

The mechanics of protein methylation have been best studied in histone modification. Histone methyltransferases are highly specific towards the nature of the amino acid residue (histone-lysine methyltransferases (E.C. 2.1.1.43) and histone-arginine methyltransferases (E.C. 2.1.1.125)) and the position of this residue in the polypeptide chain [1, 65]. Lysine residue methylation in histones is a very important element of the aforementioned "histone code" [33-36, 38]. The best characterized methylation positions in histones are Lys4 and Lys9 in the H3 histone. Besides the mentioned residues, Lys27, Lys36, Arg2, Arg17 and Arg26 residues in H3 can also be modified, as well as Arg3 in the H4 histone [33, 34, 67, 70]. 

It was demonstrated that the trimethylated Lys4 in the H3 histone is necessary for transcription activation, while dimethylated Lys4 is found both in the active and silent gene [33, 34, 70]. The heterochromatin protein 1 (HP1) interacts with the trimethylated Lys9 of H3 via its chromodomain (a recognition domain for alkylated amino acid residues), initiates local chromatin condensation, and recruits other protein factors into the assembly of an active transcription complex [26, 30, 33, 67, 70].

**Fig. 9.**
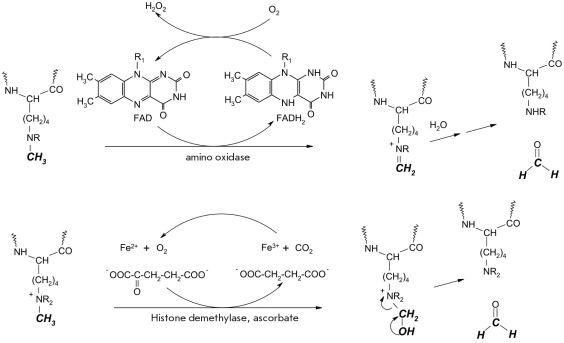
Demethylation reaction of di- and monomethylated lysine residues in histones catalyzed by the FAD-dependent aminooxidase (top), and tri-, di- and monomethylated lysine residues in histones catalyzed by histone demethylase, which functions in the presence of cofactors, Fe^2+^ ions, Alpha;-ketoglutarate and ascorbate (bottom)

Until recently, it was thought that the methylation of lysine residues was an irreversible process [1]. But a short while ago, researchers managed to extract enzymes that catalyzed the cleavage of methyl groups from lysine and arginine residues, which means that this type of posttranslational modification is also dynamic. Demethylation of lysine is an oxidative process and can be catalyzed either by the FAD-dependent polyamine oxidase, or a lysine-specific demethylase, which functions as a dioxygenase in the presence of cofactors, such as Fe^2+^ ions, Alpha;-ketoglutarate, and ascorbate (E.C. 1.5.3.4) [37, 65, 66, 82, 83]. For schematic representation of this process see Fig. 9.

**Fig. 10.**
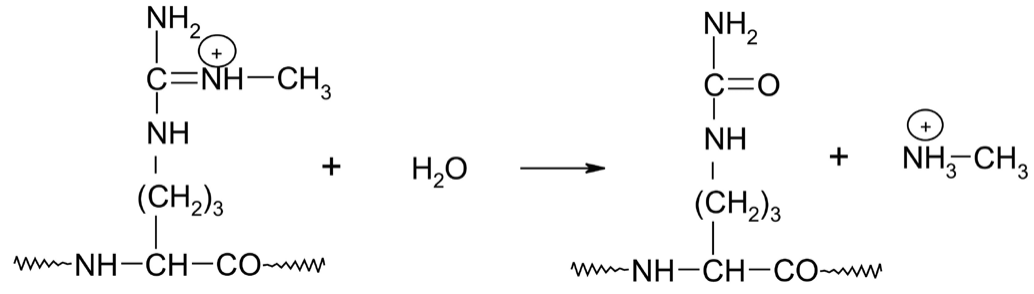
Demethylation of modified arginine residues catalyzed by the nuclear peptidylarginine deiminase (PAD4) [58]

A nuclear peptidylarginine deiminase (E.C. 3.5.3.15) can demethylate arginine residues, turning methylated arginine into citrulline [66] (Fig. 10).

Thus, methylation-demethylation and acetylation-deacetylation of specific residues in histones are major factors in gene repression and activation.

## PROTEIN PRENYLATION

**Fig. 11.**
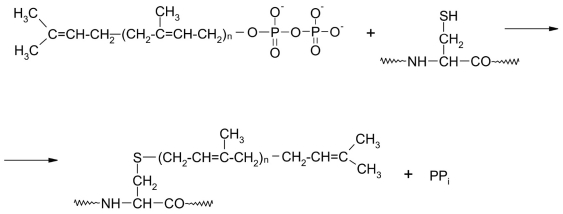
Tranfer of an isoprenoid residue from pyrophosphate to a cysteine residue in an apoprotein. n = 2 - is a farnseyl residue, n = 3 - geranyl-geranyl residue

Some cases of posttranslational modification are the addition of isoprenoid moieties onto a cysteine residue. These moieties are formed from isoprene residues - farnesyl and geranyl-geranyl (Fig. 11). Modification of proteins with these radicals is catalyzed by proteinfarnesyl and proteingeranyl-geranyl transferases, respectively (E.C. 2.5.1.58 and E.C. 2.5.1.59 or E.C. 2.5.1.60; Type I and II geranyl-geranyl transferases). Type I enzymes catalyze the transfer of a gernayl-geranyl residue onto a cysteine residue in a Cys-A-A-X sequence, while type II use the Cys-Cys-X-X, X-X-Cys-Cys or X-Cys-X-Cys sequences [47, 73-80], where A is a small aliphatic amino acid, and X are various amino acids.

**Fig. 12.**
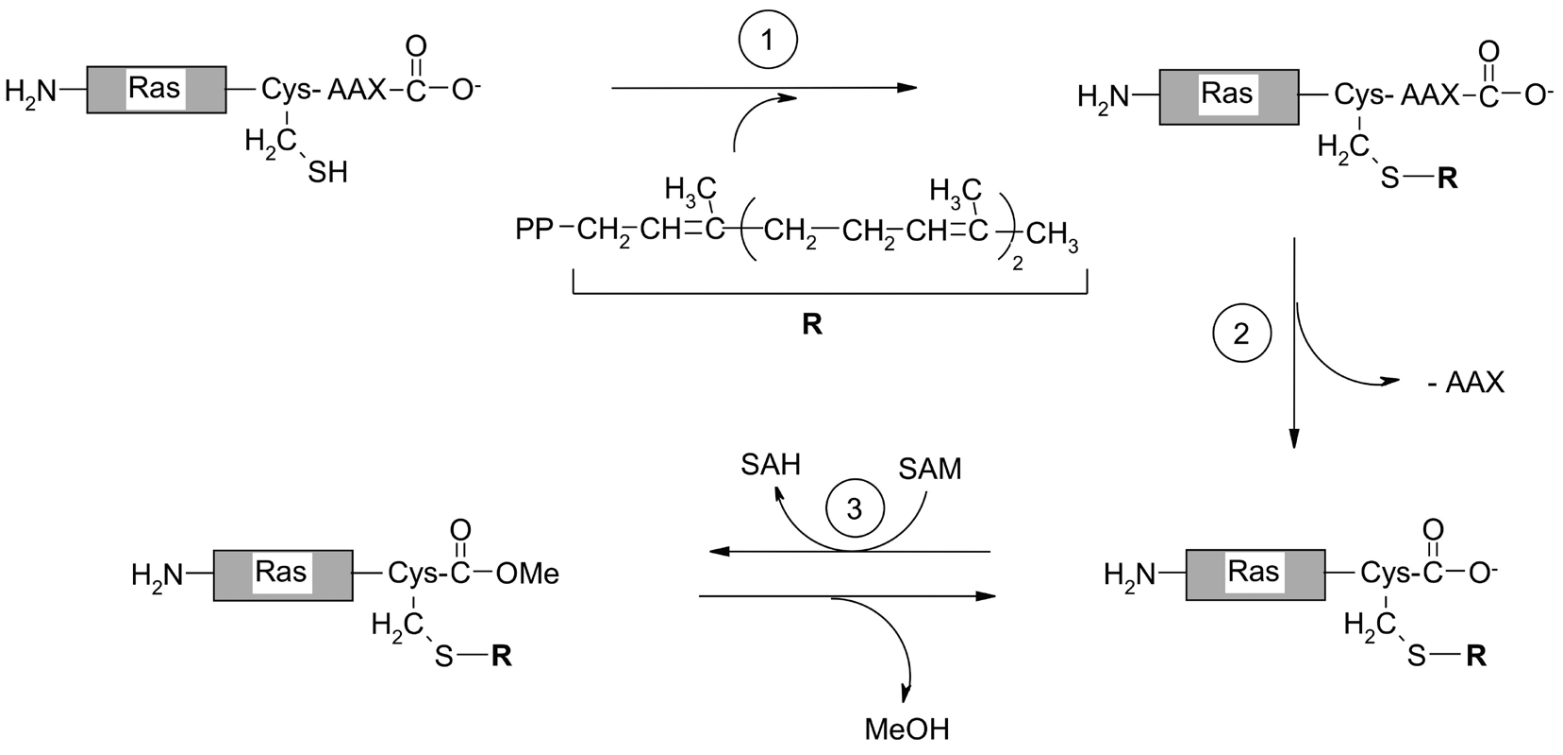
Prenylation of the Ras protein: 1 - addition of a farnesyl residue onto the Cys-A-A-X sequence (A- a small aliphatic amino acid residue, X is Leu, Phe or Met); 2 - Cleaving of the A-A-X tripeptide by the Rasconverting enzyme, which is a CysAAX-endopeptidase; 3 - carboxymethylation of the isoprenylcysteine residue catalyzed by the isoprenylcysteine carboxymethyltransferase [86]

Ras-, Rab- and Rho-family proteins (products of the ras, rab and rho proto-oncogenes, involved in cellular growth and differentiation); centromeric proteins; and γ-subunits of heterotrimeric G-proteins, chaperones tyrosine phosphotases are all subjected to prenylation [47, 73, 75, 78, 79, 81]. The C-terminal sequence of Ras-family proteins includes a Cys-A-A-X motif, in which X is the amino acid that determines the enzyme specificity: Leu, Phe, and Met in case of the type I geranyl-geranyl transferase; and Ala, Gln, Ser, Met, and Phe in the case of the farnesyltransferase [47, 74, 78, 79]. Enzymes that transfer the isoprenyl residues are metalloenzymes, and they carry a single Zn^2+^ ion for each dimeric enzyme molecule. The zinc ion activates the cysteine thiol group for nucleophilic attack by the isoprenyl moiety [73]. The addition of the isoprenyl group to the Cys-A-A-X motif is usually not the last modification of the target protein (Ras, Rho), further processing occurs via proteolytic cleavage of A-A-X tripeptide from the C-terminus by a Cys-A-A-X-specific protease; and carboxymethylation of the isoprenylcysteine residue, by the isosprenyl-cysteine-carboxymethyl transferase (E.C. 2.1.1.100) [84-87] (Fig. 12).

GTPases of the Rab family carry a Cys-Cys-X-X motif near the C-terminus. Both these cysteines can be modified by geranyl-geranyl residues with the help of type II protein geranyl-geranyl transferase, which creates two lipid anchors on the protein molecule [74, 75]. Such a protein exhibits increased affinity towards lipid membranes, and it can thus act as a unique recognition site for specific protein-protein interactions.

Proteins of the Rab family are involved in intracellular vesicle transport circulating between the cellular membrane and the cytosol. Reversible association of the protein with the cellular membrane is achieved through the isoprenyl residues decorating these proteins [75, 84].

Since 20-30% of all human oncological conditions are caused by mutations in Ras family proteins, enzymes that modify these proteins with isoprenyl residues can serve as targets for anti-tumor drugs [73, 79].

## PROTEIN GLYCOSYLATION

**Fig. 13.**
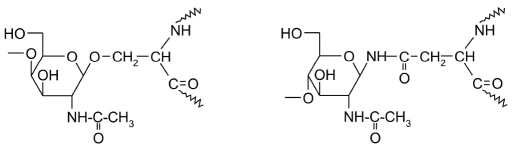
Structures of the products of N-acetylglucosamine addition onto serine and asparagine side chains in proteins

Glycosylation of proteins plays a very important role in the functioning of eukaryotic cells. Glycosylation modifies the OH-groups of serine and threonine residues (O-glycosylation) and the functional groups of asparagine residue side chains (N-glycosylation) (Fig. 13).

N-glycosylation of proteins happens at the carboxyamide nitrogen atom of an asparagine residue in the context Asn-X-Ser/Thr. N-glycoside formation begins in the endoplasmic reticulum. The oligosaccaryl transferase enzyme (E.C. 2.4.1.119) transfers a branched tetradecasaccharide fragment onto the target protein. This fragment is (Glc_3_Man_9_(GlcNAc)_2_), and it comes from the carbohydrate donor molecule dolilchol pyrophosphate.

The vast variety of glycoproteins is assured by the processing of the protein-bound tetradecasaccharide residue, which is accomplished by a set of glycosidases and glycosyl transferases.

**Fig. 14.**
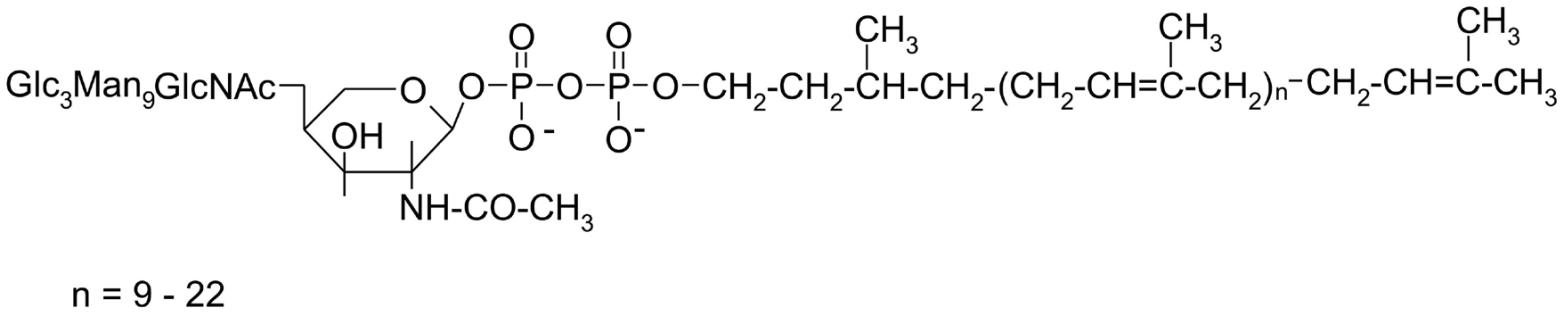
Structure of the carbohydratebearing dolichol pyrophosphate

**Fig. 15.**
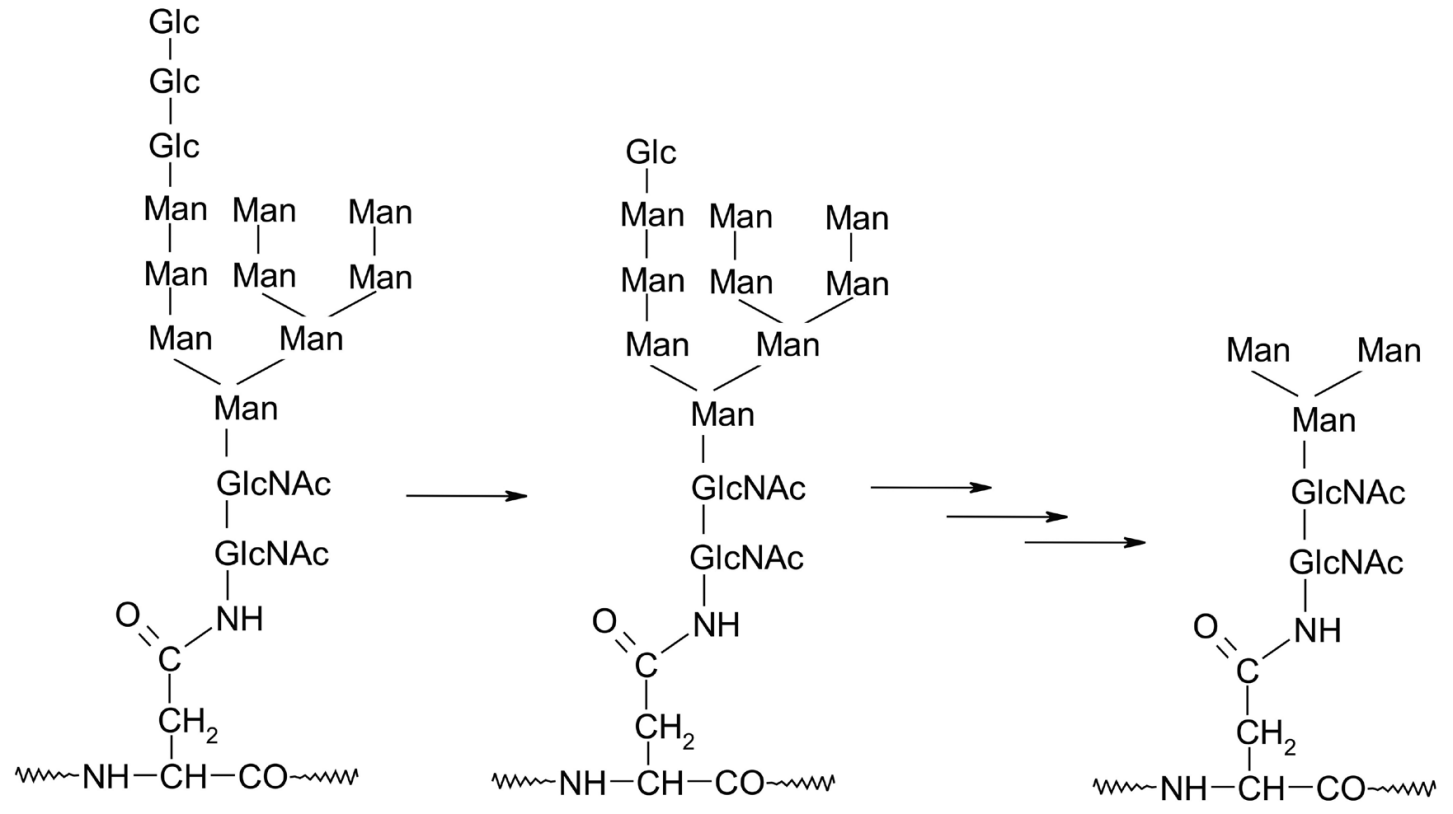
Structure and first stages of the processing of oligosaccharide fragment Glc_3_Man_9_(GlcNAc)_2_ as a part of a glycoprotein. Monosaccharides Glc - glucose, GlcNAc - N-acetylglucosamine

Figure 15 presents the structure of a bound tetradecasaccharide and the products of the first stages of processing, which are catalyzed by glucosidases I and II (E.C. 3.2.1.106) that cleave away two glucose residues, and mannosidases (E.C. 3.2.1.130) that cleave away 6 mannose residues. The glycoprotein formed after separation of the two glucose residues, and thus bearing an N-bound dodecasaccharide residue, is then recognized by the chaperones calnexin and calreticulin, which facilitate correct folding of the protein while it is being transported from the location of synthesis on the membrane-bound ribosomes to the inside of the endoplasmic reticulum [1, 88, 89, 90-93]. After a third glucose residue is cleaved away by an endoplasmic reticulum glucosidase, the chaperones lose their affinity towards the undecasaccharide and dissociate from the glycoprotein complex. UDP-glucose:glycoprotein glucosyltransferase (E.C. 2.7.8.19) returns a glucose residue back onto the undecasaccharide, which makes canexin and calreticulin continue the glycoprotein folding. This is a mechanism for maintaining the functional structure in secreted glycoproteins. 

If a glycoprotein is not folded correctly during several rounds of deglycosylation-reglycosylation, then it is transported into the cytosol. There, it is polyubiquitylated by the E3-ligase, which is a part of the degradation system for misfolded proteins in the endoplasmic reticulum and is hydrolyzed in the proteosomes [1, 88, 89, 90-94].

Correctly folded Man_9_(GlcNAc)_2_N-glycoprotein loses 6 mannose residues with the help of endoplasmic reticulum and Golgi apparatus mannosidases and forms a protein conjugated with a core pentasaccharide (Man_3_(GlcNAc)_2_). The latter can receive various monosaccharides with the help of a number of glycosyl transferases, of which there is a great many in the endoplasmic reticulum and the Golgi apparatus. Thus, the variety of glycoproteins is numbered in tens of thousands [1, 88, 89, 95].

Glycoprotein 0-glycoside chains are much shorter and simpler than N-glycoside chains. Numerous proteins, including transcription factors, nuclear pore proteins, oncoproteins, etc., contain a monosaccharide residue of N-acetylglucosamine, which is introduced into the protein by an 0-GlcNAc-transferase (E.C. 2.4.1.94) and can be cleaved by the appropriate hydrolase [1, 88, 89, 96, 97-100]. There are also di-, tri- or tetraglycoside fragment bearing 0-glycosides.

Short 0-glycoside chains in 0-glycoproteins are important for transcription activation, and they act as recognition sites during interaction with cell membrane receptors, which are involved in the transduction of signals into the cell [1, 88, 89, 100-102].

## PROTEIN SULFATION

**Fig. 16.**
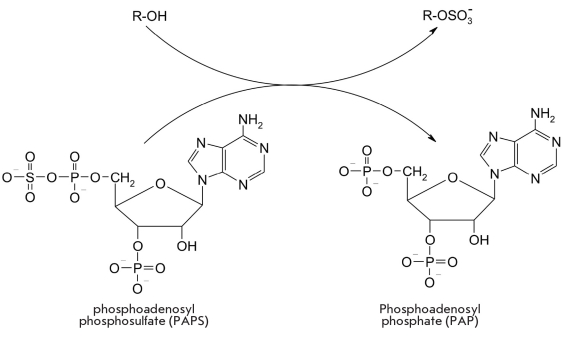
Sulfation reaction catalyzed by sulfotransferase

Another posttranslational modification of protein molecules is the addition of a sulfate residue at the OH-group of tyrosine. Phosphoadenosylphosphosulfate acts as a sulfate donor (Fig. 16). The reaction is catalyzed by the sulfotransferase enzyme (E.C. 2.8.2.20) [103, 104].

For instance, three tyrosine residues in the N-terminal region of the human chemokine cell membrane receptor (a regulator of anti-inflammatory immune reactions), which plays an important role in embryo development and in the immune response, are subject to posttranslational sulfation in the Golgi apparatus. This increases the affinity of the receptor towards its ligand, the SDF-1Alpha; chemokine. An enzyme called sulfatase (E.C. 3.1.5.6) was found in lysosomes and was able to catalyze the hydrolysis of sulfoesters [103, 105, 106].

## MONO- AND POLY(ADP-RIBOSYL)ATION

Many cellular processes, such as DNA reparation, apoptosis, and the functioning of the spindle during cell division, use mono- and poly(ADP-ribosyl)ation as an important regulating mechanism [107]. Various pathogenic bacteria secrete toxins that ADP-rybosylate human proteins, thus causing severe diseases, such as cholera, diphtheria, pertussis, and botulism [108-111].

**Fig. 17.**
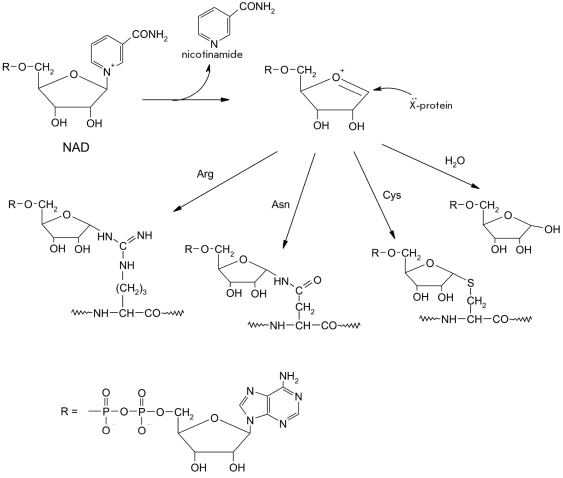
(ADPribosyl) ation of nucleophilic amino acid residues (X) present in the protein (cysteine, arginine and asparagine) [1]

NAD^+^ acts as a donor of the ADP-ribosyl residue. The positively charged nicotinamide bond is cleaved by the ADP-ribosyltransferase (E.C. 2.4.2.31) and forms a ribo-oxocarbene cation, which interacts with various nucleophilic groups in protein active sites and leads to their (ADP-ribosyl)ation (Fig. 17) [108, 109].

For instance, pertussis toxin transfers the created cation to the thiolate chain of a cysteine residue in the active site of the human G_i_-protein Alpha;-subunit. This protein regulates synthesis of the second messenger cAMP [1, 111, 112]. Cholera toxin transfers an ADP-ribosyl residue onto the arginine residue in the human G_s_-protein Alpha;-subunit ([1, 111, 113]. The ADP-ribosyl residue can also be transferred by the C3 toxin of Clostridium botulinum onto the nucleophilic Asn41 residue of the minor GTPase of the Rho protein superfamily, which leads to actin depolymerization and impairment of the metabolic processes of the host cell [1, 111].

Diphtheria toxin ADP-ribosylates His715 in the eEF-2 elongation factor and, therefore, blocks the translocation of peptides on ribosomes and the whole translation process in human cells [114].

**Fig. 18.**
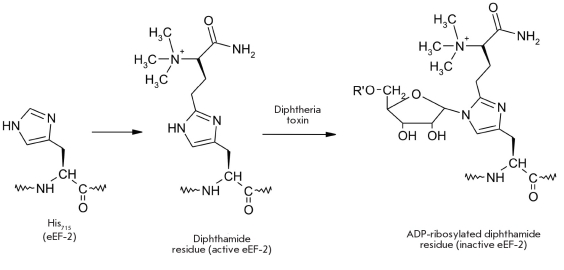
Modification of the His715 residue in the structure of the human eEF-2 elongation factor results in the blocking of protein synthesis in human cells

In reality, His715 is subjected to stepwise complex modification: first, an aminocarboxypropyl residue is transferred from S-adenosylmethionine (SAM), then SAM-dependent N,N,N-trimethylation takes place, then the carboxyl group is amidated in a glutamine-mediated fashion, thus forming a diphthamide residue, and only then does the toxin ADP-ribosylate the diphthamide residue at the N3 atom of the imidazole ring (Fig. 18) [115-117].

**Fig. 19.**
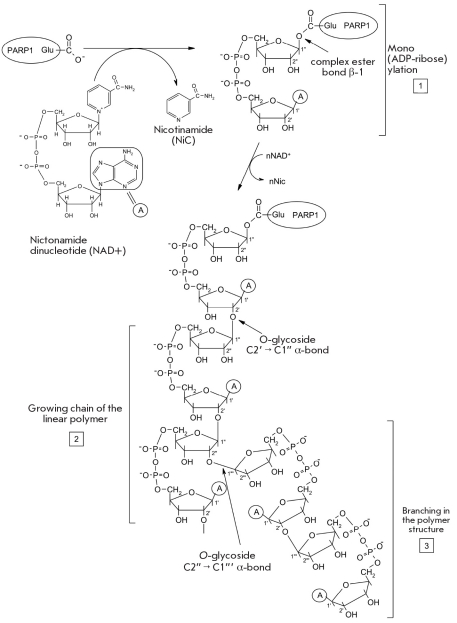
Mechanism of poly(ADP-ribose) synthesis in the self-modification of PARP1

During the lifetime of the organism, the genome constantly suffers the effects of genotoxic agents of both exogenic and endogenic nature [118]. An approximate estimate demonstrated that every day the genomes of human cells experience up to 104-106 instances of DNA damage [119]. Under these circumstances, the stability of cell genome is one of the most important factors in maintaining the survival of a multicellular organism, since any uncorrected damage to DNA can promote the emergence of mutator cell phenotypes [120]. Poly(ADP-ribose) (PAR) synthesis is one of the immediate reactions of the cell in response to DNA breaks under the influence of ionizing radiation, or alkylating or oxidizing agents [121, 122]. This process is catalyzed by enzymes poly(ADP-ribose)polymerases (PARPs), which are constantly and abundantly expressed in the cell [123]. PARPs are activated in response to DNA breaks and catalyze the posttranslational modification of a range of DNA-binding proteins by covalently adding a polymer poly(ADP-ribose) to the carboxyl groups of glutamic and aspartic acid in the acceptor proteins [124]. Currently, approximately 30 nuclear proteins that are poly(ADP-ribosyl)ated in vivo and in vitro have been described [123, 125]. All these proteins exhibit DNA-binding activity and are involved in DNA metabolism (replication, transcription, reparation) or in chromatin formation (histones). Several enzymes of the poly(ADP-ribose) polymerase class have been found in eukaryotes, including PARP1, PARP2, and PARP3, which have nuclear localization; tankyrases 1 and 2, which interact with telomere proteins and are thought to regulate telomere function; VRAP (193 kDa), found in cytoplasmic ribonucleprotein vault-particles [126]; sPARP - a truncated form of PARP1, which does not require activation by DNA breaks [127]; and macro PARPs (BAL/PARP-9, PARP14, PARP15), which are involved in the epigenetic modification of chromatin [124, 128]. Ninety percent of the nuclear poly(ADP-ribose) synthesis is caused by PARP1 activity [129]. This protein is expressed at a constant level throughout the cell cycle, and each cell carries around 1.0·10^6^ of the protein molecules, which amounts to 1 protein molecule for each 6000 nucleotide pairs [130]. Catalytically inactive PARP1 is present in the nucleoplasm and is activated by DNA breaks. It then binds to the damaged area and catalyzes PAR synthesis [128]. PARP synthesizes poly(ADP)-ribose in three stages: initiation, elongation, and branching of the polymer (Fig. 19).

The first stage involves the formation of the ester bond between the ADP-ribose and the carboxyl group in a glutamate residue in the acceptor protein [131, 132]. The second stage involves the formation of an 0-glycoside bond between the C2' and C1'' atoms of the ADP-ribose, thus creating a linear polymer of ADP-ribose molecules [133, 134]. In the third stage, the glycoside bond links the C2'' and C1''' atoms of the ADP-ribose, forming branches in the polymer structure [135, 136] (Fig. 19).

The rate of the chemical reaction at the mono(ADP-ribosyl)ation stage is approximately 200 times slower than at the elongation stage [137]. Based on the measurement of kinetic parameters of the in vitro PARP-catalyzed poly(ADP-ribosyl)ation reaction, the authors of [138] hypothesize that the latter reaction is inter-molecular, meaning that PARP1 functions as a homodimer at the DNA break site. Two molecules react with the DNA break at once, and during the reaction both molecules simultaneously synthesize PAR and function as acceptors. The covalent modification of PARP1 by the addition of a charged poly(ADP-ribose) residue leads to alterations in the enzyme's physicochemical characteristics and its dissociation from the DNA-complex [139]. Thus, regulation of PARP1 DNA-binding activity can be achieved through self-modification [140].

Discovery of poly(ADP-ribosyl)ation modifications in chromatin remodeling proteins, histones in vivo, and topoisomerases in vitro leads to the assumption that PARP1 is involved in chromatin remodeling during DNA repair [123, 133, 141]. It was demonstrated that the kinetic parameters of DNA repair reactions were influenced by the presence of histones on the damaged DNA [123]. In vivo poly (ADP-ribosyl)ation of the H1 histone and the histones forming the nucleosome core during DNA damage can play an important role in DNA repair, especially if the DNA is structured as chromatin, since histone modification can lead to their dissociation from the DNA molecule, thus allowing the repair enzymes easy access to the damaged site [123, 140].

Therefore, the current overall notion is that the cell response to damaged DNA can be modulated by the activity of PARP1. On one hand, PARP1 activates repair processes, thus promoting cell survival; on the other hand, when DNA damage is irrepairable and the emergence of a mutator phenotype is highly probable, "overactivation" of PARP1 induces cell death [142]. This is why the PAR synthesis catalyzed by PARP in the process of interacting with DNA breaks can be regarded as a signal of the DNA damage level, which is used to determine the cell's future functional strategy.

## OXIDATION OF THE SULFOHYDRIDE MOIETY OF THE CYSTEINE RESIDUE IN PROTEINS

A large number of proteins are characterized by the formation of disulfide bonds in a reaction between cysteine residues either inside a single polypeptide chain or between different polypeptide molecules. Such bonds fulfill a structural function and determine the tertiary and quaternary structure of the protein, which are vital for the protein's metabolic functions in the organism. This modification is also involved in the regulation of the cell's reduction-oxidation status, which affects numerous aspects of cellular processes, such as proliferation, differentiation, and apoptosis by changing the functioning of proteins via a reversible modification of cysteine residues [143-147].

Oxidation of cysteine residues involves the following processes: formation of a disulfide bond, the formation of sulfi- and sulfoacids, and binding of glutathione [145]. Formation of a disulfide bond is accomplished via the oxidation of the electron-rich sulfhydryl moeity (or of the thiolate anion, which is generated from the former after proton dissociation) of the cysteine residue side chain. One-electron oxidation of the sulfhydryl moiety leads to the formation of a thiyl radical, which can dimerize into a disulfide [147].

**Fig. 20.**
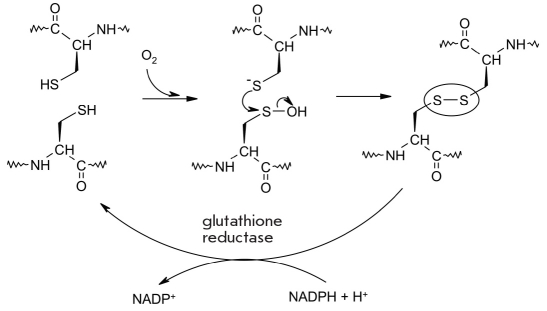
Oxidation of the sulfhydryl group of the cysteine residue, which can he reduced again into a thiol group with the help of NAD(P) H and a glutathione reductase [147]

Under physiological conditions, most of the sulfhydryl groups are in oxidized form and thus involved in disulfide bonds. Reduction of the disulfide bonds in vivo is accomplished by the glutathione tripeptide γ-Glu-Cys-Gly (GSH), which converts into oxidized glutathione (GSSG). High levels of NAD(P)H and of the glutathione reductase (E.C. 1.8.1.7) and thioredoxinreductase (E.C. 1.8.1.9) enzymes lead to the reduction of oxidized glutathione [143-147] (Fig. 20). As proteins move down the secretory pathways of eukaryotic cells, the levels of glutathione and NAD(P)H decrease, which is why most proteins exist in structures stabilized by disulfide bonds [148].

Oxidizing agents (hydrogen peroxide, hydroxide radical) can oxidize the cysteine sulfhydryl group into the cysteine-sulfenic acid (-SOH) [147]. Interaction of the cysteine-sulfene acid residue with the closest Cys-S^-^ group also results in the formation of the disulfide bond.

Reduction of the disulfide bond can result either from thiol-disulfide exchange with either glutathione or thioredoxin (TSH), a low-moleclar-weight (12 kDa) protein which contains catalytically active sulfhydryl groups in its active center (Cys-Gly-Pro-Cys) and plays the central role in the regulation of the reduction-oxidation status of disulfide bonds in proteins, which in turn governs a wide range of cellular processes. Oxidized forms of these compounds are reduced by NAD(P)H and glutathione reductase/thioredoxin reductase [146-149].

**Fig. 21.**
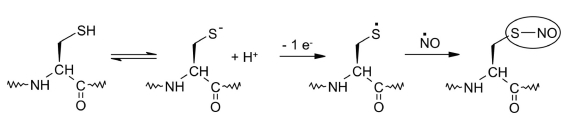
Oxidation of the thiolate ion in the presence of nitrogen oxide results in the formation of cysteinyl-nitroxide [154]

Both the thiolate ion and the thiyl radical can interact with other oxidizing agents and radicals (such as NO^•^) (Fig. 21). The resulting CysSNO molecule is involved in oxidation signaling in the cell [150-154].

## HYDROXYLATION OF PROTEIN FUNCTIONAL GROUPS

**Fig. 22.**
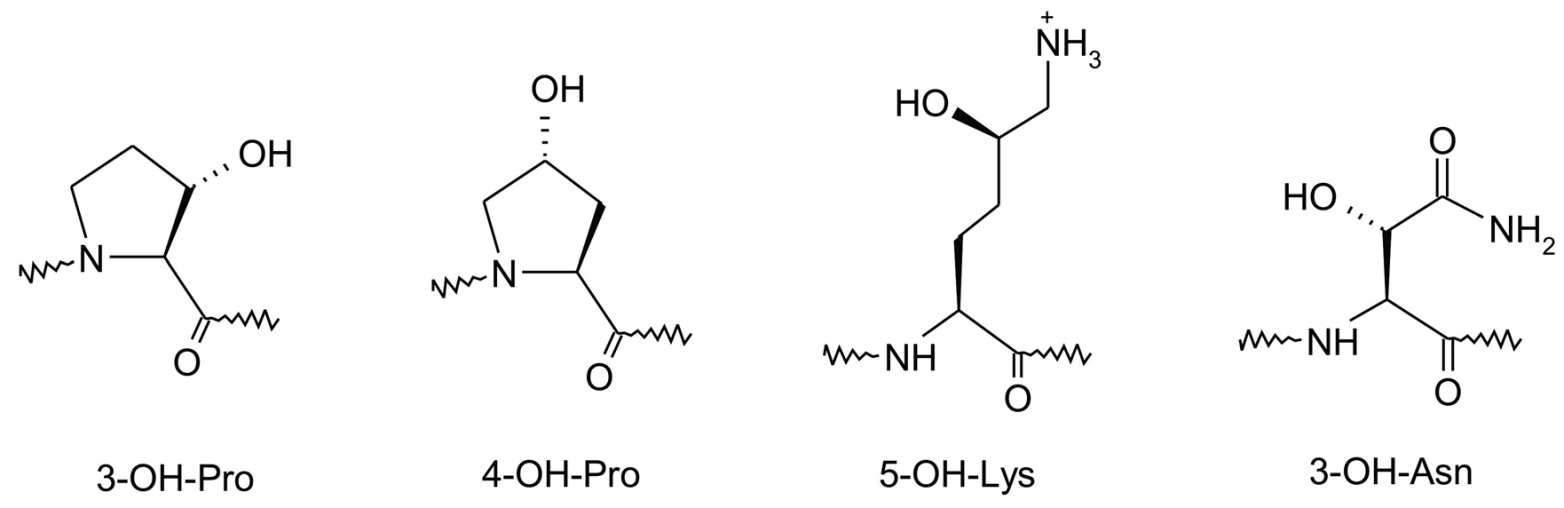
Structure of monooxygenated proline, lysine, and asparagine residues

Another type of posttranslational modification is the oxidative hydroxylation reaction. This reaction takes place at non-nucleophilic amino acid residue side chains: the CH_2_-groups of proline, lysine and asparagine form 3-hydroxyproline, 4-hydroxyproline, 5-hydroxyproline, and 3-hydroxyasparagine, and this process is catalyzed by iron-containing monooxygenases of the E.C. 1.14.16 subclass [155, 156, 157] (Fig. 22).

Oxidized proline and lysine residues play an important role in the formation of hydrogen bonds in the tri-strand spatial structure of the connective tissue protein collagen. Oxidation takes place at the Pro-Gly and Lys-Gly sequences. 4-hydroxyproline is found about 10 times more often than 3-hydroxyproline [155-160].

Besides the above said, hydroxylation of specific amino acid residues plays a role in the function of the HIF transcription factor (hypoxia inducible factor) [156, 159-161]. This protein is activated under conditions of insufficient oxygen. It induces the transcription of a wide range of genes, including the gene encoding erythropoietin, which stimulates erythrocyte differentiation from precursor cells, thus increasing the transport of oxygen to cells suffering from hypoxia [160].

The Alpha;-subunit of the human HIFAlpha;β is posttranslationally hydroxylized in the central region of the molecule at two proline residues, Pro402 and Pro564, forming 4-OH-Pro, and also in the C-terminal region at Asn803, forming 3-OH-Asn [156]. A molecule bearing hydroxylized proline residues is subjected to ubiquitylation by the E3 ligase, and the lifespan of HIF is determined by the rate of hydroxylation, ubiquitylation, and proteolysis in the proteasomes. Low 0_2_ pressure causes slow hydroxylation of proline. High oxygen pressure causes the Pro-hydroxylase to efficiently hydroxylize Pro residues, which increases affinity towards the E3 ligase 1 000-fold and causes rapid ubiquitylation and decay in the proteasomes, while at low oxygen pressures, HIF is fairly stable and can exist for a long time [162, 163].

**Fig. 23.**
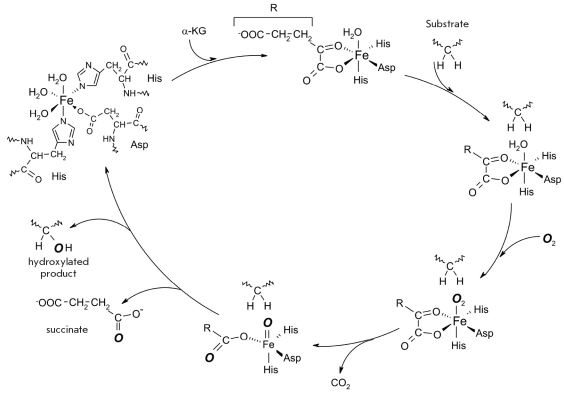
Mechanism of the hydroxylation reaction

The hydroxylation of proline and asparagine side chains is catalyzed by a family of oxygenases that contain non-heme iron [163]. The active site of the enzyme (Fig. 23) contains two histidines and one asparagine, which take up three of the six coordination spaces around the Fe^2+^ atom, while two spaces are occupied by the Alpha;-ketoglutarate co-substrate; and the sixth, by oxygen. Interaction of the Alpha;-ketoglutarate and oxygen results in oxidative decarboxylation and yields CO_2_ and succinate, which accepts one of the oxygen atoms of the molecular oxygen. The second oxygen atom takes part in the generation of the high-valence Fe^4+^=0 complex. The latter group is an effective oxidizing agent, which cleaves the unactivated C-H bond at the C3 or C4 atom of proline, C5 of lysine and C3 of asparagine, thus forming •C-H and Fe^3+^-OH radicals.

Transfer of the hydroxyl radical •OH from Fe^3+^-OH to •C-H results in the hydroxylation of the amino acid side chain, which by itself is not a donor of electrons and does not act as a nucleophile in this reaction. Monooxygenases, which catalyze hydroxylation reactions, attach the hydroxide radical in a stereospecific manner.

## POSTTRANSLATIONAL CARBOXYLATION OF THE GLUTAMIC ACID RESIDUE

Most protein factors, which are involved in blood clotting in mammals, contain several residues of γ-carboxyglutamic acid (Gla). This residue appears in blood clotting factors as a result of posttranslational modification; namely the fixation of CO_2_ by the γ-methylene carbon atom of glutamic acid (Glu) during the factor's progress down the secretion pathways [164-166]. The Gla residue side chain, which bears two negatively charged carboxyl groups, has a capacity to form chelate complexes with bivalent cations, which is especially important for interaction with the Ca^2+^ ion [164].

Gla can be found in such proteins as prothrombin and blood clotting factors IX and X, which are proenzymatic forms of proteases [164]. Carboxylation of 10-12 Glu residues in the N-terminal region of the proenzymes in a sequence of up to 40 amino acids leads to the binding of several Ca^2+^ ions and to conformation alteration of the blood clotting factors, which then associate on the surface of platelets adjacent to the proteases, which activate the factors by partial proteolysis and initiate the blood clotting cascade [164-166].

**Fig. 24.**
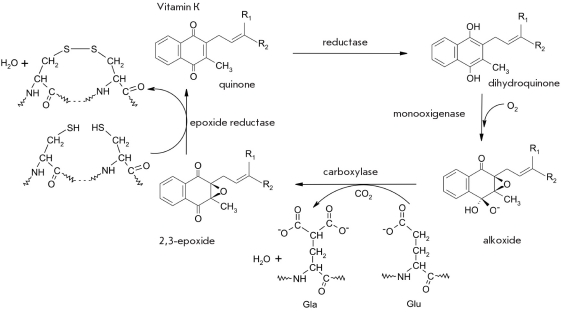
Vitamin K-dependent carboxylation of a glutamic acid residue catalyzed by γ-glutamylcarboxylase. The 2,3-epoxide of vitamin K is reduced by vitamin K 2,3-epoxide reductase

Carboxylation of the glutamic acid residue is catalyzed by the γ-glutamilcarboxylase (E.C. 1.14.99.20), which uses the reduced (dihydronaphtochinol) form of vitamin K (Fig. 24) [1, 164-166]. The oxidation of the reduced form of vitamin K by oxygen results in the formation of a hyperperoxide adduct of vitamin K, which forms a cyclic alkoxide anion, 2,3-epoxide of vitamin K, and generates a strong base, which captures a proton from the γ-methylene carbon atom of glutamic acid. The formed carbanion attacks the carbon atom of CO_2_ and forms a new C-C bond in the malonyl side chain of the Gla residue. Reduction of the 2,3-epoxide of vitamin K into its original form is catalyzed by 2,3-epoxydereductase (E.C. 1.1.4.1), which is associated in a complex with protein disulfide isomerase in the endoplasmic reticulum (E.C. 1.8.4.2) [167].

## NON-ENZYMATIC MODIFICATION OF FUNCTIONAL GROUPS IN PROTEINS | PROTEIN GLYCATION

**Fig. 25.**
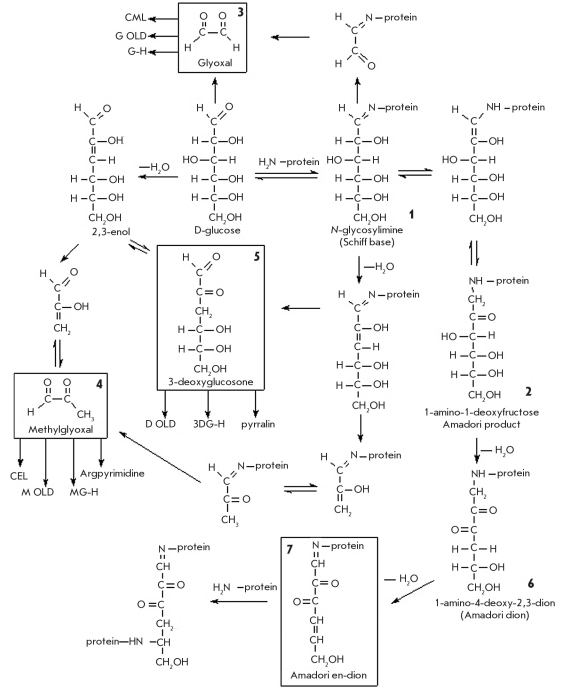
Glycation of proteins in the presence of D-glucose. The rectangles show the main precursors of AGEs, which are formed during glycation

Protein glycation is an endogenous non-enzymatic addition of reducing sugar residues present in the bloodstream to the side chains of either lysine or arginine residues in proteins. A schematic representation of the glycation process, which can be divided into the early and late stages, is shown on Fig. 25. The first stage of glycation involves the nucleophilic attack of the glucose carbonyl group by an ε-amino group of lysine or a guanidine moiety of arginine, which results in the formation of a labile Schiff base - N-glycosylimine (1). The formation of the Schiff base is a relatively rapid and reversible process [168]. Next, the glycosylimine regroups and forms an Amadori product, 1-amino-1-deoxyfructose (2). This process happens more slowly than the formation of glycosylimine, but much quicker if compared to the rate of Schiff base hydrolysis. This is why proteins bearing 1-amino-1-deoxyfructose residues tend to accumulate in blood. Modification of lysine residues at the early glycation steps is thought to be facilitated by the close proximity of histidine or lysine residues, which catalyze this process [169].

**Fig. 26.**
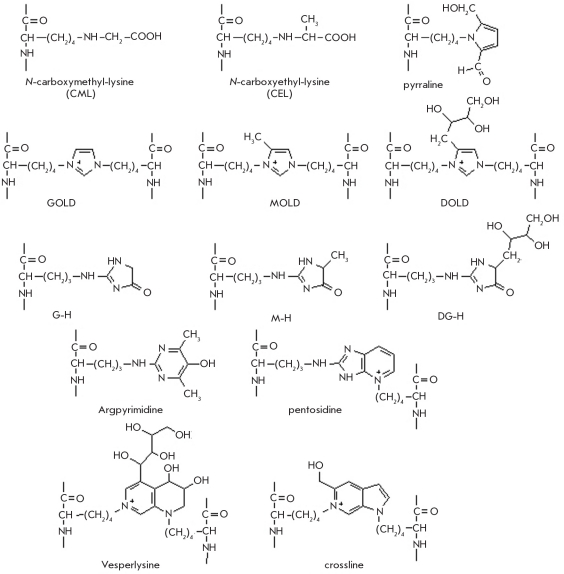
Structure of certain AGEs formed as a result of in vivo protein modification by D glucose

The late stage of glycation, which involves transformations of the N-glycosylimine and the Amadori product, is a slower and less studied process. It results in the formation of stable, advanced glycation end-products (AGEs) (Fig. 26). There are published data [170] on the direct involvement of α-dicarbonyl compounds in AGE formation (glyoxal (3), methylglyoxal (4), and 3-deoxyglucosone (5)). These compounds form in vivo both during glucose degradation and in the transformations of the Schiff base during the modification of lysine resides in proteins by glucose (Fig. 25).

Reactions between α-dicarbonyl compounds and the ε-amino groups of lysine residues or the guanidinium groups of arginine in proteins result in the formation of protein crosslinks, which lead to complications caused by the protein glycation seen in diabetes and other diseases. Moreover, sequential dehydration of the Amadori product results in the formation of a 1-amino-4-deoxy-2,3-dion (6) and en-dion (7) at the C4 and C5 atoms, respectively (Fig. 25). These side chains can form intra- and intermolecular protein crosslinks [170].

Some AGEs have been characterized, including N_ε_-carboxymethyl-lysine (CML) and N_ε_-carboxyethyl-lysine (CEL) [171], bis(lysyl)imidazole adducts (GOLD, MOLD and DOLD) [172], imidazolones (G-H, MG-H and 3DG-H) [173, 174], pyrraline [175], argpyrimidine [176], pentosidine [177], crossline [178], and vesperlysine [179] (Fig. 26)]. Among these pentosidine, crossline and vesperlysine are fluorophores, and their fluorescence emission maximum (λ_em_ = 440 nm) is shifted into the long-wave region, compared to tryptophan residue fluorescence in proteins [180]. This property of AGEs allows to monitor the glycation reaction progress by measuring the fluorescence at the excitation wavelength characteristic of the forming fluorophore glycation products (glucophores).

## INTRAMOELCULAR POSTTRANSLATIONAL AUTOCATALYTIC CYCLIZATION

A very impressive type of posttranslational modification is the autocatalytic restructuring of the peptide backbone in the folded protein during GFP (green fluorescent protein) maturation. This protein is encoded by a single gene, and the chromofore is made up of three amino acid residues, Ser65-Tyr66-Gly67, capable of posttranslational autocatalytic cyclization, which does not require any cofactors or substrates [181-183].

**Fig. 27.**
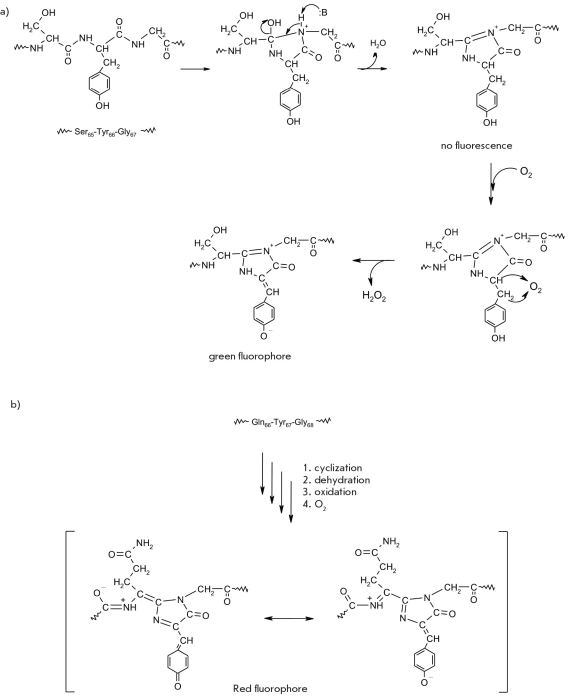
Formation of (a) green and (b) red chromophores in proteins from tripeptides by intramolecular posttranslational autocatalytic cyclization

Formation of the chromophore requires that the precursor take on the form of a β-barrel. This folded and colorless GFP-precursor bears the Ser65-Tyr66-Gly67 tripeptide in a spatially squeezed conformation in which the amide of Gln-67 can attack the peptide carbonyl and form a pentatomic tetrahedral adduct (Fig. 27, a). Then, this adduct is dehydrated, and the stable cyclic intermediate product slowly autooxidizes, forming a double bond coupled to the phenol ring of Tyr-66. This last oxidation reaction produces a chromofore with an excitation maximum of 506 nm.

GFP is used as an in vivo vital marker, which allows the study of various processes taking place in live cells and organisms [184-186]. Fusion proteins based on GFP are used in novel drug screenings [187, 188], apoptosis detection [189], in the visualization of chromosome dynamics [190], and in many other applications [191, 192]. Several volumes of Methods in Enzymology [193] and Methods in Cell Biology [194] are dedicated to GFP. The discovery of fluorescent genetic markers was awarded the Nobel Prize in 2008. 

During the last decade, the number of studies with other colored proteins similar to GFP but extracted from coral has been steadily growing [195-197]. A drawback of these proteins is their marked propensity to aggregate, which however can be rectified by mutagenesis [198]. A schematic representation of the formation of a red fluorophore from the Gln66-Tyr67-Gly68 tripeptide in a protein molecule is shown in Fig. 27, b.

## PROTEIN HOMOCYSTEINYLATION 

**Fig. 28.**
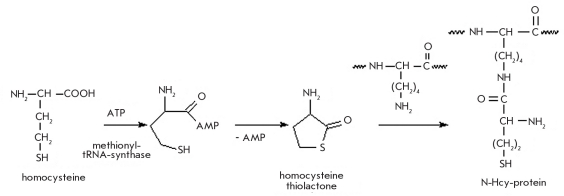
N-homocysteinylation of proteins by the homocysteine thiolactone

The majority of methylation processes in live organisms use S-adenosylmethionine, thus forming S-adenosylhomocysteine. The latter is hydrolyzed by the adensylhomocysteinase (E.C. 3.3.1.1) enzyme into adenosine and homocysteine. This reaction catalyzed by methionyl-tRNA synthetase (E.C. 6.1.1.1) turns homocysteine into thiolactone (this is a side reaction for this enzyme) [199]. Homocysteine thiolactone is an acylating agent and can react with the functional groups of lysine residues [200-203]. The ε-amino group of lysine performs a nucleophilic attack of the carbonyl carbon atom of the thiolactone, which results in decyclization of the lactone and the formation of an additional sulfhydryl moeity (Fig. 28).

This type of modification is characteristic of blood proteins (albumin, hemoglobin, transferring, and globulins) [204-207]. Ninety percent of the homocysteine in human blood plasma is incorporated into N-homocysteylated serum albumine (HSA) [201]. It is known that the main HSA homocysteinylation site both in vitro and in vivo is the Lys-525 residue [208]. Furthermore, two additional albumin modification sites were discovered at Lys-4 and Lys-12 [209].

**Fig. 29.**
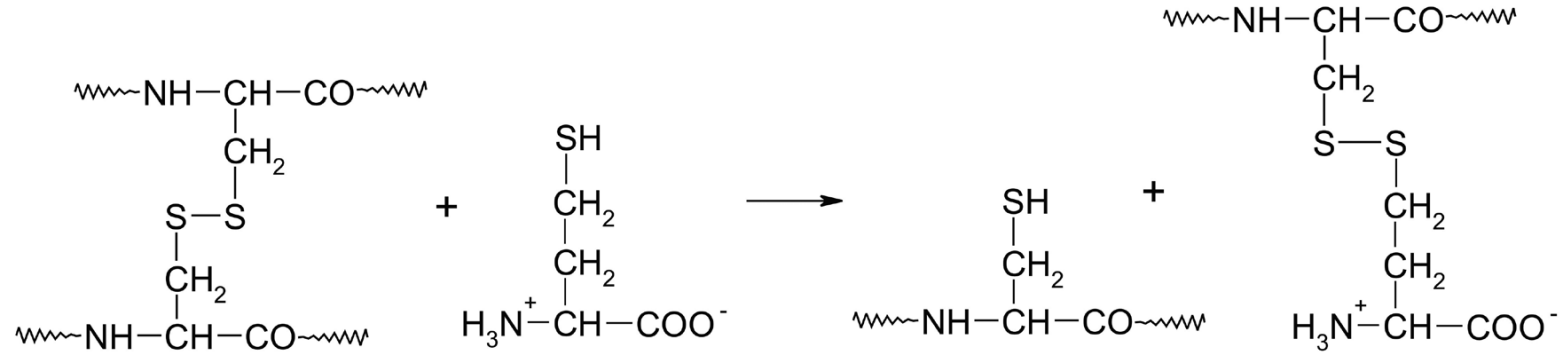
Protein S-homocysteinylation

Homocysteine can take part in disulfide exchange reactions with S-S bonds in proteins, thus forming S-homocysteinylated proteins (Fig. 29) [200, 202, 206, 207, 210].

Homocysteinylation of proteins has a considerable effect on their biological activity, including increased sensitivity to oxidation and increased propensities for oligomerization, denaturation, and sedimentation. The introduction of 8-9 homocysteine residues into the methionyl-tRNA-synthase and 11-12 residues into trypsin completely deactivates these proteins [207]. N-homocysteinylation of human serum albumin lowers its RNA-hydrolyzing activity considerably [205]. Multiple homocysteinylation of cellular proteins can eventually result in cell apoptosis [200, 201, 203, 206, 210].

## DEAMIDATION AND TRANSAMIDATION

**Fig. 30.**
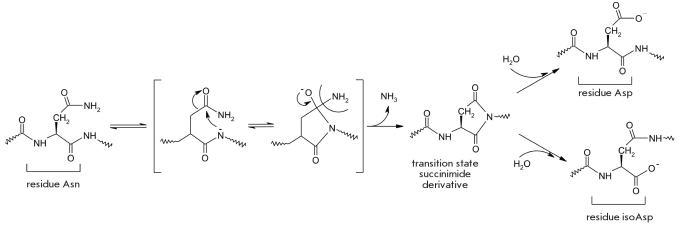
Deamidation of asparagine residues in peptides and proteins at pH>5

One of the types of posttranslational modification, which plays an important role in cellular functions, is the deamidation of the amides of dicarbonic acids. Many authors believe these reactions to be non-enzymatic cleavage of ammonia from the amide group of asparagine or glutamine, resulting in an intermediate product, a cyclic imide (Fig. 30) [211-215]. The rate of this product's formation is determined by the local amino acid surroundings and the characteristics of the solution (pH and ingredients) [213, 214]. Asparagine residues in proteins are deamidated 40 times more often than glutamine residues. Furthermore, the rate of asparagine deamidation is 100-fold greater than the rate of glutamine deamidation [214].

The cyclic imide decays forming either aspartate residue, which forms in the largest quantities (3:1), or an isoaspartate residue, in which the peptide bond involves the β-carboxyl group of the aspartate side chain [216, 217]. In the latter case, the length of the protein increases by one methylene group (CH_2_), which can influence the structure and the functioning of the protein, including its stability [214, 216, 217].

Deamidation reactions result in the formation of an ionizable carboxyl group charged negatively under physiological conditions, which alters the overall charge of the protein molecule and its spatial structure [214].

The β-iospeptide bond formed by lysine and glutamine side chains is considered by the organism to be an aberration of a normal peptide bond, which is formed by the Alpha;-amino groups and carboxyl groups of amino acids, and is corrected by the protein isoaspartyl-O-methyltransferase (PIMT) (E.C. 2.1.1.77), a widespread cellular enzyme [211, 212, 216]. The deamidation reaction of Asn/Gln and a deficit of PIMT cause serious illnesses in humans, such as cataract [218], Alzheimer's disease [219], autoimmune diseases [220], and prion-dependent encephalopathy [214, 221, 222]. 

According to Robinson's hypothesis, the instability of the asparagine and glutamine residues in cellular proteins under physiological conditions determines a key biological function, which is a programmed biological clock mechanism limiting the lifespan of proteins and peptides [212, 223, 224].

Deamidation, as well as ADP-ribosylation, can be caused by bacterial toxins. The cytotoxic necrotic factor 1 from Escherichia coli (CNF1) and the dermonecrotic toxin (DNT) from Bordetella deamidate small GTPases in the human organism, such as Rho A (Gln63), Rac1, and Cdc42 (Gln61), which results in blockage of GTP hydrolysis and disorders in the regulation of cytoskeleton remodeling [225-228].

**Fig. 31.**
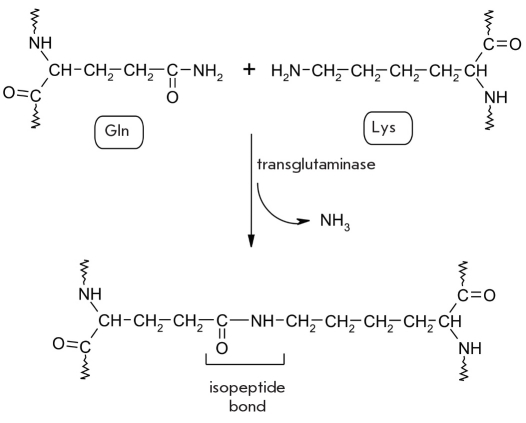
Transamidiation catalyzed by transglutaminase (E.C. 2.3.2.13)

Deamidation is often coupled with subsequent transamidation (interaction of the ε-amino group of a lysine residue with the side chain of a glutamine residue in the same protein molecule), which is one of the types of crosslinks characteristic of posttranslational modification (Fig. 31) [228-232].

This process leads to the formation of multiple bonds between glutamine and lysine residues in protein molecules, which results in a massive protein aggregate whose subunits are cross-linked. This is an important process in the metabolism of skin and hair and also during the healing of wounds [233]. 

## Acknowledgements

This work on the effects of chemical modification of human serum albumin on the RNA-hydrolytic activity of the protein was performed with support from the Interdisciplinary Integration Project for Basic Research Siberian Branch RAS №88 and the Russian Foundation for Basic Research (grant №09-04-01483a).
